# Handgrip strength predicts leg power but not cardiorespiratory fitness in children

**DOI:** 10.3389/fphys.2025.1645483

**Published:** 2025-08-14

**Authors:** Salem Alotaibi, Rizwan Qaisar, M. Azhar Hussain, Osama Aljuhani, Maha H. Alhussain, Diogo Luís Marques, Daniel Almeida Marinho, Shaea A. Alkahtani

**Affiliations:** ^1^ Exercise Physiology Department, College of Sport Sciences and Physical Activity, King Saud University, Riyadh, Saudi Arabia; ^2^ Department of Basic Medical Sciences, College of Medicine, University of Sharjah, Sharjah, United Arab Emirates; ^3^ Space Medicine Research Group, Research Institute of Medical and Health Sciences, University of Sharjah, Sharjah, United Arab Emirates; ^4^ Cardiovascular Research Group, Research Institute of Medical and Health Sciences, University of Sharjah, Sharjah, United Arab Emirates; ^5^ Department of Finance and Economics, College of Business Administration, University of Sharjah, Sharjah, United Arab Emirates; ^6^ Department of Social Sciences and Business, Roskilde University, Roskilde, Denmark; ^7^ Department of Physical Education, College of Sport Sciences and Physical Activity, King Saud University, Riyadh, Saudi Arabia; ^8^ Department of Food Sciences and Nutrition, College of Food and Agricultural Sciences, King Saud University, Riyadh, Saudi Arabia; ^9^ Department of Sport Sciences, Faculty of Human and Social Sciences, University of Beira Interior, Covilhã, Portugal; ^10^ The Research Center in Sports Sciences, Health Sciences and Human Development, Covilhã, Portugal

**Keywords:** handgrip strengh, long jump, muscular power, children, health and performance

## Abstract

**Purpose:**

The current study examined the ability of handgrip strength (HGS) to predict leg muscle power, cardiorespiratory fitness, and movement behaviors in children.

**Methods:**

One hundred eighteen male children aged 10–13 years from primary and middle schools in Saudi Arabia were recruited for this cross-sectional study. Physical fitness tests included HGS, standing long jump (SLJ), and a 20-m shuttle run test (20mSRT). Daily movement behaviors (time spent on physical activity, sedentary activities, and sleep) were measured for seven consecutive days using accelerometers. A multiple linear regression analysis was conducted to analyze the ability of HGS to predict SLJ distance and 20 m SRT laps. Age and body mass index were also included in the model as covariates.

**Results:**

HGS was significantly correlated with SLJ distance (*r* = 0.44, *p* < 0.05), 20mSRT (*r* = 0.22, *p* < 0.05), sedentary time (*r* = 0.34, *p* < 0.05), and moderate to vigorous physical activity time (*r* = −0.36, *p* < 0.05). In the regression models, HGS had a significant positive effect on predicting SLJ distance (*β* = 2.64, *p* < 0.001). Age was found to be a significant predictor of 20mSRT (*β* = 3.65, *p* < 0.001).

**Conclusion:**

These findings highlight that HGS may serve as a simple and practical indicator of musculoskeletal fitness, aiding early detection of functional limitations and informing physical development strategies in children Saudi boys, suggesting that HGS may be used to predict SLJ distance in this population.

## Introduction

Reference values for physical fitness have been widely proposed for use among children and adolescents. For example, the literature highlights the physical fitness norms of children from various international and regional data, including Germany (Golle et al., 2015), Australia (Catley and Tomkinson, 2013), Greece (Tambalis et al., 2016), China (Zhang et al., 2021), and Saudi Arabia (Alqahtani et al., 2023). In this regard, Eurofit tests include nine tests of physical fitness. A population-based systematic review study presented the norms and centiles of Eurofit tests, which is the most representative study in the field (Tomkinson et al., 2018). Generally speaking, most physical fitness components increase with age during childhood, except cardiorespiratory fitness, which slightly decreases, particularly among girls (Hoffmann et al., 2019; Sagat et al., 2023). In line with most studies, the Saudi study (Alqahtani et al., 2023) showed the role of age on muscular fitness among Saudi children. Several factors, including daily lifestyle and body composition, are among the limitations of most of these reference values and norms.

Anthropometry and lifestyle are important factors that can explain variations in physical fitness parameters. An extensive Greek study emphasized that abdominal adiposity, rather than general obesity, is a strong predictor of poor physical fitness (Arnaoutis et al., 2018). The study also found that a sedentary lifestyle was associated with low physical fitness during childhood (Arnaoutis et al., 2018). Height and weight have advantages in certain physical fitness tests and disadvantages in others. For example, being overweight negatively affects performance in the Progressive Aerobic Cardiovascular Endurance Run (PACER) test and the standing long jump (SLJ) test, whereas tall and heavy children perform better in the handgrip strength (HGS) test (Müller et al., 2022). Obesity was associated with handgrip strength, but this association was not observed with other physical fitness tests in Hong Kong Chinese schoolchildren (Yip et al., 2022). These interactions of confounders could play a significant role in the relationship between muscular strength and power, as some factors favor strength but may limit muscular power.

Strength underpins a combination of morphological and neural factors (Suchomel et al., 2018) and can contribute to an increase in muscular power. It is worth noting that most studies examining the relationship between HGS and muscular power were among athletes and adults. For example, there were significant correlations between HGS and vertical jump test, horizontal jump test, and curl-ups test in college students, with body mass index (BMI) affecting these relationships (Vaidya and Nariya, 2021). Another study on college students also found a significant relationship between HGS and SLJ (Trosclair et al., 2011). Unlike adults, the relationship between HGS and muscular power in childhood could be different and requires further exploration. Interestingly, Cohen et al. (2014) reported a significant correlation between HGS and cardiorespiratory fitness among male children (r = 0.42). A similar correlation between HGS and aerobic capacity was also reported in sedentary young females (Dag et al., 2021). HGS is a marker of muscular strength, but it is also used as an ideal predictive marker of cardiovascular health (Ramírez-Vélez et al., 2016). Additionally, HGS has been recognized as a biomarker of general health, nutritional status, and a potential predictor of future cardiometabolic risk in pediatric populations, reflecting underlying neuromuscular development and physical resilience. Thus, examining the association between HGS and cardiorespiratory fitness will reflect the overall view of health-related physical fitness and the physical growth and improvement in children (Matsudo et al., 2014).

The relative value of muscle and strength to fat or BMI has been proposed and used in the prediction equation to better estimate physical fitness in children. For example, the muscle-to-fat ratio was correlated with cardiorespiratory fitness, and HGS to BMI was correlated with different physical fitness parameters in Spanish schoolchildren (Manzano-Carrasco et al., 2023). Likewise, HGS relative to BMI showed significant correlations with most fitness tests among Greek children (Llagjeviq-Govori et al., 2024). In most cases, age, gender, and body composition are the primary elements used in predictive equations of physical fitness tests, including the 20-m shuttle run test (20mSRT), HGS, and SLJ (Mendoza-Muñoz et al., 2020; Blanchard et al., 2018). However, there is a lack of studies analyzing these relationships among Saudi Arabian children, indicating that further research is necessary to understand the ability of upper body strength to predict performance during horizontal jump tests and cardiorespiratory endurance tests. Thus, we aim to address both performance and health perspectives by examining the predictive value of HGS on lower limb function (a marker of performance) and aerobic fitness (a health-related indicator) in school-aged children.

Therefore, based on the research gaps, the current study aimed to examine the ability of HGS to predict performance in the SLJ and 20mSRT tests, as well as movement behaviors, among Saudi Arabian children. The models also analyzed the influence of age and BMI on predicting physical performance. We hypothesized that HGS would be a significant predictor of leg muscle power, as assessed by the SLJ test, but not of cardiorespiratory fitness. We also anticipated that higher HGS would be associated with favorable movement behavior profiles.

## Methods

### Participants

All participants were male children aged 10–13 years from primary and middle schools in Taif City, Saudi Arabia. Taif City has 4 main branch offices (South, North, East, and West offices). Six schools (3 primary schools and 3 middle schools) were aimed to be included in the study. Schools were selected randomly, and formal letters were sent to the schools requesting their cooperation with the study’s procedure. Invitation letters were sent to approximately 120–150 parents at each school. In total, approximately 180 were affirmative, averaging about 30 responses per school, with the lowest response rate being 16 from one school. However, 20 of the respondents could not be reached, mainly due to unanswered phone calls. Permission and consent for participation were obtained from the participants’ families/parents. Ethical approval was obtained from the Ethical Review Committee at King Saud University (IRB: KSU-HE-24-234). G*Power software (V.3.1.9.7) was used to calculate the required sample size to obtain 95% confidence with a margin of error of 5%, and a sample size of at least 99 participants was required. Twenty-three out of 160 students did not participate because of their absence on the scheduled testing days. Moreover, 15 out of 137 students who were examined returned accelerometry devices that either contained no data or did not meet the minimum required number of recording days or hours. Six out of 122 students were excluded during the analysis because they were outliers or had missing data in the regression. Data from 118 children who completed the study were entirely analyzed. Exclusion criteria included adolescents, adults, and older adults, with metabolic or mobility diseases, and an inability to complete the requirements of the study.

### Study design and procedure

The study was a cross-sectional design. All tests were conducted in the morning between 8:00 and 10:00 a.m. on school days. Anthropometry and body composition were measured. Physical fitness tests included HGS, SLJ, and 20mSRT, which were performed on the school’s sports field. There was a minimum of one researcher or research assistant for every 10 participants during the 20mSRT. Moreover, daily movement behaviors, including physical activity, sedentary behavior, and sleep, were measured for all children using accelerometers worn for seven consecutive days.

## Materials

### Anthropometry measures

Heights and weights were measured using a portable stadiometer scale device (Seca 770, Seca, Germany). BMI was calculated by dividing the weight in kilograms by height in squared meters, based on the CDC Global reference data (Cole et al., 2000). Participants were classified as underweight, normal weight, overweight or obese according to the International Obesity Task Force (IOTF) criteria.

### Physical fitness tests

Physical fitness included cardiorespiratory fitness, handgrip strength, and standing long jump tests, and the procedure of these tests has been published elsewhere (Alghamdi et al., 2025). For clarity, we briefly describe the tests procedure as below.

#### Cardiorespiratory fitness assessment

Cardiorespiratory fitness was assessed using a modified version of the 20mSRT. The test follows a standardized, recorded instruction from the PACER CD (Meredith and Welk, 2010). Participants were instructed to run forth and back over the marked 20 m distance in rhythm with the audible signal. The test speed starts at 8.5 km/h, with a speed increment of 0.5 km/h every minute, representing a stage. The test is ended either at the point of individual volitional exhaustion or when participants fail to maintain the required running speed twice (De Miguel-Etayo et al., 2014). Performance is quantified as the total number of shuttles completed, and the classification of aerobic fitness level reported by Tomkinson et al. (2018) was used.

#### Handgrip strength assessment

HGS was assessed using an isometric handgrip dynamometer (Baseline® Smedley spring-type dynamometer, Fabrication Enterprises, Inc., White Plains, NY, United States). The dynamometer was adjusted accordingly to accommodate differences in participant hand size and ensure a comfortable grip position. To motivate participants to have their maximal effort, they were given verbal encouragement. The participant performed two trials using the dominant hand. If the second trial was significantly higher than the first, a third trial was performed, and the highest score was recorded in kg.

#### Standing long jump assessment

The SLJ test examines the strength of the lower limbs. The participant stands in a line at the starting point, bends his knees, and tries to jump, pushing his body forward as much as possible. The connection between the heel and the ground is marked, and the distance from the starting point is measured in centimeters. The participant was given two trials, and the highest score was recorded. Performance is quantified as the distance jumped in cm, and the levels of performance are classified based on the reference values reported by Tomkinson et al. (2018).

#### Data treatment of physical fitness tests

All fitness data were extracted and treated in Excel (Microsoft Office 2019, United States). The participants’ fitness levels were categorized according to a previous sex-specific and age-specific normative centile ranks (Tomkinson et al., 2018). For this study, participants below the 40th centile were classified as low; 40–60th were classified as moderate, and those above 60th centile were classified as high.

### Accelerometry of daily movement behaviors

ActiGraph wGT3X-BT triaxial accelerometers (ActiGraph LLC, Pensacola, FL, United States) were used to measure the 24-h movement behaviors, including sleep, sedentary time, light physical activity (LPA), and moderate-to-vigorous physical activity (MVPA). The participants wore the accelerometer on their non-dominant wrist for 24 h across seven consecutive days. Accelerometer data were initialized and downloaded using ActiLife software (version 6.13.6, ActiGraph LLC, Pensacola, FL). Data were downloaded and saved in raw format (GT3X) and subsequently converted to CSV format to facilitate processing of the raw data. Accelerometer data were processed in R (http://cran.r-project.org) using the package GGIR (version 4.3.0). Participants were excluded if their accelerometer wear time was less than 16 h per day for at least 3 days (Rowlands, 2018). Published non-dominant wrist ENMO cut-points of <35.6 mg, at least 35.6 mg and less than 201.4 mg, and ≥ 201.4 mg were used to estimate sedentary time, LPA, and MVPA, respectively (Hildebrand et al., 2017; Hildebrand et al., 2014).

### Data analysis

With a sample size of n = 118 Saudi boy respondents, a statistical approach to the analysis was appropriate. Descriptive and inferential analysis were conducted, and HGS adjusted for BMI (HGS/BMI) was calculated. A multiple linear regression analysis was conducted to analyze the ability of handgrip strength to predict SLJ distance and 20mSRT laps. Age and body mass index were also included in the model as independent variables. The assumptions underlying the applied ordinary least squares (OLS) regression model were assessed and found to be satisfactorily met. Specifically, linearity was confirmed; Homoscedasticity was found using graphical plots; Normality of residuals was confirmed by histograms and Shapiro-Wilk normality tests; No perfect multicollinearity was found for the covariates, and no severe degree of multicollinearity issues were detected after assessing VIF measures. The estimated regressions were:

(1) Standing *Long Jump (cm)* = *β*_0_ + *β*_1_∙*Age* + *β*_2_∙*HGS* + *β*_3_∙*BMI* + *ε*
(2) 20-m *Shuttle Run (laps)* = *β*_0_ + *β*_1_∙*Age* + *β*_2_∙*HGS* + *β*_3_∙*BMI* + *ε*

Where HGS represents handgrip strength and *β*_2_ represents the increase in SLJ and 20mSRT laps (different effects for the two) when HGS increases by 1. The 5% significance level was applied in all analysis. The unstandardized β-coefficients are reported and interpreted, while the standardized β-coefficients are available from the authors upon request. Statistical software Stata 18.0 SE Standard Edition was used for all statistical analyses (Stata Statistical Software, Release 18; College Station, TX: StataCorp LLC).

## Results

Table 1 presents correlation coefficients of HGS, HGS adjusted for BMI (the HGS/BMI ratio) with various physical and behavioral factors. HGS significantly positively correlates with age (correlation coefficient r = 0.58, and p-value p < 0.05) (older children on average have higher HGS), BMI (r = 0.35, p < 0.05) (higher BMI is associated with higher HGS), SLJ (r = 0.43, p < 0.05) (higher SLJ is associated with higher HGS), 20mSRT laps (r = 0.22, p < 0.05) (more 20mSRT laps are associated with higher HGS), and sedentary time (r = 0.34, p < 0.05) (more sedentary time is associated with higher HGS), while it significantly negatively correlates with MVPA (r = −0.36, p < 0.05) (more MVPA is associated with lower HGS). These outcomes of sedentary and MVPA should be read with caution as we did not examine confounders or possible measurement artifacts. On the other hand, it could be patterns of movement behaviors among children, which require further examination.

**TABLE 1:** Relationships between HGS and HGS/BMI with shuttle run laps, long jump, sleep, sedentary, LPA, and MVPA. Correlation coefficients.

	Age (years)	BMI (kg/m^2^)	Long jump (cm)	Shuttle run laps (count)	Sleep (min/day)	Sedentary (min/day)	LPA (min/day)	MVPA (min/day)
HGS	0.58*	0.35*	0.43*	0.22*	−0.17	0.34*	−0.14	−0.36*
HGS/BMI	0.49*	−0.40*	0.59*	0.49*	0.03	0.19*	−0.16	−0.28*

*p < 0.05.

HGS: handgrip strength; BMI: body mass index; LPA: light physical activity; MVPA: moderate to vigorous physical activity.

HGS/BMI shows significantly positive correlations with age (r = 0.49, p < 0.05), long jump (r = 0.59, p < 0.05), shuttle run laps (r = 0.49, p < 0.05), and sedentary time (r = 0.19, p < 0.05), while it is negatively associated with BMI (r = −0.40, p < 0.05), MVPA (r = −0.28, p < 0.05), and LPA (r = −0.17, p > 0.05), (Table 1). Sleep does not show a significant correlation with either HGS or HGS/BMI. This suggests that HGS and HGS/BMI are linked to age and physical performance - particularly long jump and shuttle run laps. A higher BMI is associated with greater HGS; however, its negative correlation with HGS/BMI indicates that excess weight may not contribute to relative strength. Increased sedentary time is linked to higher HGS, whereas higher MVPA is associated with lower HGS and HGS/BMI, potentially reflecting differences in muscle adaptation. The lack of a significant relationship with sleep suggests that sleep duration do not play a significant role in determining HGS or HGS/BMI.

**FIGURE 1:**
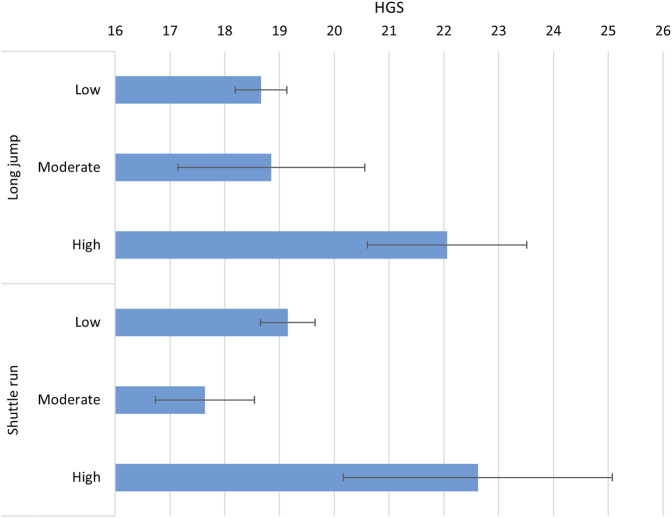
Average values of handgrip strength for Saudi children based on the European standard reference of long jump and shuttle run laps (Tomkinson et al., 2018).

Figure 1 shows the relationship between HGS and various performance levels in SLJ and 20mSRT laps, using the European reference values for these two tests. A clear pattern emerges, where Saudi boys with higher performance in both SLJ and 20mSRT, tend to have greater HGS. The error bars indicate uncertainty of the estimates–there is some overlap, but the general increase in HGS from low to high categories is clear.

Table 2 presents regression analyses examining the effects of age, HGS, and BMI on long jump and shuttle run laps. The model for long jump explains 37.7% of the variance (R^2^ = 0.377), while the model for shuttle run laps accounts for 34.0% of the variance (R^2^ = 0.340). For the long jump, HGS has a significantly positive effect (*β* = 2.64, p < 0.001), indicating that higher HGS is associated with greater jump distance–when HGS increases by 1 kg, long jump on average increases by 2.64 cm. Age also has a positive coefficient (*β* = 3.12), but it is not statistically significant. BMI, however, has a significant negative effect (*β* = −2.85, p < 0.001), suggesting that higher BMI is linked to shorter long jump distances. For shuttle run laps, age is a significant positive predictor (*β* = 3.653, p < 0.001), showing that older individuals tend to complete more laps. HGS does not show a significant association (*β* = 0.47, p > 0.05), suggesting that HGS does not directly predict shuttle run performance–but it is significant at the 10% level (p = 0.063) when shuttle run laps increase by 1 lap, shuttle run laps increase by 0.467 laps. BMI has a significant negative association with shuttle run laps (*β* = −1.51, p < 0.001), indicating that higher BMI is associated with lower performance in this endurance-based test. The regressions suggest that both age and HGS contribute positively to long jump performance, while a higher BMI had negative effects on long jump and shuttle run performance. Shuttle run laps appear to be more influenced by age than by HGS, highlighting potential differences in the physical demands of power-based versus endurance-based activities.

**TABLE 2:** Regressions for long jump and shuttle run laps.

	Long jump	Shuttle run laps
Age	3.12	3.65***
HGS	2.64***	0.47
BMI	−2.86***	−1.51***
Constant	100.1***	−2.08
N	118	118
R^2^	0.37	0.34

*p < 0.05, **p < 0.01, ***p < 0.001.

HGS: handgrip strength; BMI: body mass index.

β reflects cm/laps increase per one unit in the covariate, e.g., 1 kg increase in HGS increases long jump by 2.64 cm, respectively, increases the number of shuttle run laps by 0.47.

**FIGURE 2:**
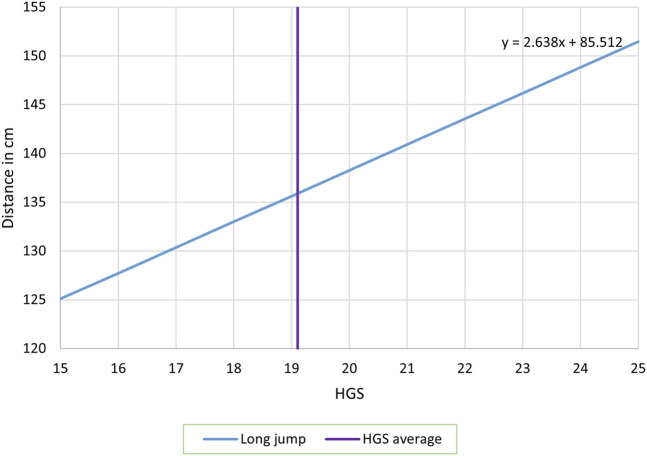
The estimated regression equation of the standing long jump (distance in cm), using handgrip strength (HGS) in kg, and controlled for age and body mass index.

The relationship between HGS and long jump is illustrated in Figure 2, where we see that higher HGS is associated with higher levels of long jump (highly significant). Particularly, we notice that at the average level of HGS (vertical purple line at 19.11), the long jump reaches its average level as well (135.92 cm).

## Discussion

We investigated the ability of HGS to predict SLJ and 20mSRT performance, as well as daily movement behaviors, in 10- to 13-year-old Saudi boys. Our data indicates a concordance between HGS and lower limb strength. Additionally, we found associations between the levels of physical activity and HGS. Conversely, the amount of sleep did not exhibit a significant association with HGS in Saudi boys.

HGS was positively associated with age, SLJ, and the number of laps completed in the 20mSRT. The positive association with age indicates the ongoing growth process that drives an increase in muscle mass and strength during the pre-adolescence stage (Dodds et al., 2014). Interestingly, we observed divergent associations between BMI and HGS, as well as HGS relative to BMI. While BMI was positively correlated with HGS, it exhibited a negative correlation with HGS/BMI. This observation suggests that while boys with higher BMIs tend to have greater absolute HGS, the increase in strength might not be proportional to their body mass. When adjusted for BMI, the relative strength (HGS/BMI) was lower in boys with higher BMIs, implying that excess weight may not contribute to functional strength relative to body size in this age group.

Participants with higher HGS exhibited superior performance in the SLJ and 20mSRT laps compared to those with lower HGS. The physical tasks of the SLJ and the 20mSRT heavily rely on the optimal functioning of the lower limb muscles. Thus, our observations suggest a potential concordance between the strengths of the upper and lower limb muscles. This observation aligns with previous findings of a positive association between upper and lower limb performances during the growth phase (Milliken et al., 2008). Similar observations are also reported in advanced age (da Costa Pereira et al., 2024), suggesting a potential coupling between the health of upper and lower limb muscles.

Upper body strength is known to contribute significantly to activities that require explosive lower limb power, such as the SLJ, as well as agility and speed, such as the 20mSRT. Thus, the positive correlation observed between SLJ performance and HGS indicates that the ability to generate force with the hands and arms may contribute to the overall momentum and stability required for a successful jump. These observations align with previous reports that the HGS appears as a covariate of impulsive abilities, such as jumping and running, in physically active individuals (Cronin et al., 2017). Notably, when HGS was adjusted for BMI, its association with SLJ and 20mSRT performance became even stronger. This result suggests that the inherent force production capacity of skeletal muscle primarily drives the relationship between upper and lower limb strength. This observation is supported by recent findings in pre-adolescent children, which indicate that muscle mass and strength can be somewhat independent (Leite et al., 2024), highlighting the role of muscle quality in determining strength.

We found positive correlations of HGS and HGS/BMI with the time spent in a sedentary lifestyle. A sedentary lifestyle is known to cause muscle weakness and atrophy in adolescents (Oh et al., 2025), which contradicts our findings. It is possible that the boys with a higher baseline HGS might be more inclined towards a sedentary lifestyle without experiencing a significant drop in HGS. Additionally, other lifestyle, nutritional, and growth-related variables may have influenced the associations between HGS and HGS/BMI and sedentary activities. However, these boys exhibited superior physical fitness, as demonstrated by their long jump and shuttle run records, suggesting that sedentary activities did not negatively impact their physical fitness at this period of life. It should be noted that we did not examine confounders. For example, in the study of the association between sitting time and HGS, confounders among adults include age, body mass index, alcohol intake, cigarette smoking, resistance exercise, aerobic physical activity, household income, education level, hypertension, diabetes mellitus, dyslipidemia, and depression (Lee et al., 2020). Further investigation with a large sample size and consideration of confounders is warranted.

We found negative associations between HGS and HGS/BMI with MVPA, which is contradictory to the established literature (Oh et al., 2025). This observation may reflect the differences in the types of physical activities by the study participants. While MVPA is generally beneficial for overall health and fitness, it is possible that activities categorized as MVPA in this study did not specifically target or promote HGS development compared to other forms of exercise, such as resistance training (Kozlenia et al., 2024). In addition, a recent study shows that only vigorous physical activity exhibits positive associations with HGS in developmental stages (Moliner-Urdiales et al., 2010). Thus, categorizing MVPA into moderate and vigorous physical activities may have clarified the associations between physical activity levels and HGS. These findings suggest that HGS, a simple and inexpensive test, may be used in school or clinical settings to screen for deficits in lower limb muscle power. This is particularly important in the early identification of children at risk of impaired functional development or reduced participation in physical activity, with implications for both health promotion and athletic training programs.

A key strength of this study lies in its use of standardized tools for measuring physical fitness and movement behaviors. HGS is a well-established indicator of upper body strength and overall physical fitness (Vaishya et al., 2024). Furthermore, the SLJ and 20mSRT effectively assess the functional capacity of lower limb muscles (Cronin et al., 2017). The study participants had a homogeneous ethnic, racial, and geographic background, minimizing several confounding factors associated with genetics, climate, and lifestyle. However, it is essential to acknowledge some limitations of this study. The cross-sectional design prevents the establishment of causal relationships. Additionally, the sample was limited to boys in a specific age range and geographical location, which may limit the generalizability of the findings to other populations. We did not investigate the types of physical activities or the specific mechanisms by which physical activities are connected to HGS. Moreover, movement behaviors were classified using validated wrist acceleration cut-points developed in small study populations of young children (aged 7–11 years, n = 30). These cut-points reflect absolutes rather than participants-specific relative intensity, which may have led to some misclassification of movement behaviors (Fairclough et al., 2021). Wrist acceleration cut-points may underestimate absolute mean sedentary time compared to posture-based methods (Suorsa et al., 2020).

Our findings provide valuable insights into the relationship between HGS and physical performance in 10- to 13-year-old Saudi boys. The findings suggest that upper body strength, as measured by HGS, is positively associated with lower limb power. Additionally, we report a general concordance in the strength development of upper and lower limbs during this developmental stage. These findings emphasize the importance of considering upper body strength in assessments of physical fitness and in the design of interventions aimed at promoting physical activity in young boys. Future studies should investigate the mechanistic associations between upper and lower body strength, with relevance to lifestyle and physical activities.

## Data Availability

The raw data supporting the conclusions of this article will be made available by the authors, without undue reservation.
